# Activation of the Kinin B1 Receptor by Its Agonist Reduces Melanoma Metastasis by Playing a Dual Effect on Tumor Cells and Host Immune Response

**DOI:** 10.3389/fphar.2019.01106

**Published:** 2019-09-25

**Authors:** Andrea Gutierrez Maria, Patrícia Dillemburg-Pilla, Marina de Toledo Durand, Elaine Medeiros Floriano, Adriana Oliveira Manfiolli, Simone Gusmão Ramos, João Bosco Pesquero, Clara Nahmias, Claudio M. Costa-Neto

**Affiliations:** ^1^Department of Biochemistry and Immunology, Ribeirão Preto Medical School, University of São Paulo, Ribeirão Preto, Brazil; ^2^Department of Medicine, University of Ribeirão Preto, Ribeirão Preto, Brazil; ^3^Department of Pathology, Ribeirão Preto Medical School, University of São Paulo, Ribeirão Preto, Brazil; ^4^Department of Biophysics, Federal University of São Paulo, São Paulo, Brazil; ^5^INSERM U981, Department of Molecular Medicine, Gustave Roussy Cancer Center, Villejuif, France

**Keywords:** melanoma, des-Arg^9^-bradykinin, B1 receptor, immune response, inflammation, tumor microenvironment, metastasis

## Abstract

Metastatic melanoma is an aggressive type of skin cancer leading half of the patients to death within 8–10 months after diagnosis. Kinins are peptides that interact with B1 and B2 receptors playing diverse biological roles. We investigated whether treatment with B1 receptor agonist, des-Arg^9^-bradykinin (DABK), has effects in lung metastasis establishment after melanoma induction in mice. We found a lower number of metastatic colonies in lungs of DABK-treated mice, reduced expression of vascular cell adhesion molecule 1 (VCAM-1), and increased CD8^+^T-cell recruitment to the metastatic area compared to animals that did not receive treatment. To understand whether the effects of DABK observed were due to the activation of the B1 receptor in the tumor cells or in the host, we treated wild-type (WT) and kinin B1 receptor knockout (B1^−/−^) mice with DABK. No significant differences in the number of melanoma colonies established in lungs were seen between WT and B1^−/−^mice; however, B1^−/−^mice presented higher VCAM-1 expression and lower CD8^+^T-cell infiltration. In conclusion, we believe that activation of kinin B1 receptor by its agonist in the host stimulates the immune response more efficiently, promoting CD8^+^T-cell recruitment to the metastatic lungs and interfering in VCAM-1 expression. Moreover, treatment with DABK reduced establishment of metastatic colonies by mainly acting on tumor cells; hence, this study brings insights to explore novel approaches to treat metastatic melanoma targeting the B1 receptor.

## Introduction

Metastatic melanomas are very resistant to standard chemotherapies with a survival time of 8–10 months after being diagnosed with stage IV melanoma (Eggermont et al., 2014). Due to lack of effective treatment, extensive efforts have been made to develop new therapeutic approaches (Shah and Dronca, 2014; Maverakis et al., 2015). Targeted therapies such as BRAF and MEK inhibitors, epigenetic modification, and immunotherapy or anti-programmed cell death 1 are the most current methods that have revolutionized metastatic melanoma treatment (Herzberg and Fisher, 2016). Kinins are peptides that belong to the kallikrein–kinin system (KKS), playing biological roles such as vasodilation, inflammatory response, and stimulation of immune cells *via* activation of the G-protein-coupled receptors (GPCRs), B1 and B2 (Schulze-Topphoff et al., 2008). While B2 receptor is constitutively expressed in a variety of cell types, B1 receptor expression is low or not detected under physiological conditions, although studies suggested that the central nervous system and immune cells constitutively express B1 receptor (Wotherspoon and Winter, 2000; Ma and Heavens, 2001; Dutra, 2017).

Mice with tumors derived from TM5 melanoma cells previously activated with B1 receptor agonist, des-Arg^9^-bradykinin (DABK), presented smaller tumors and increased animal survival (Dillenburg-Pilla et al., 2013). Moreover, primary melanomas developed in B1 receptor knockout (B1^−/−^) mice showed a higher number of mitotic cells and increased activation of proliferative pathways (Maria et al., 2016). Despite the evidences that B1 receptor plays a protective role during melanoma growth, studies have demonstrated that kinin receptors are highly expressed in other types of tumors and their activation could contribute to cancer growth and migration (da Costa et al., 2014). Nevertheless, it is still unknown whether the effects observed in these studies could be due to B1 receptor activation or due to a cross talk between B1 and B2 receptors (Barki-Harrington et al., 2003).

To understand whether activation of B1 receptor could play a role in lung metastasis of melanoma, the established B16F10 murine melanoma cell line model was used. We induced lung metastasis in mice and treated with DABK. Animals that received DABK had a lower number of melanoma colonies established in the lungs, decreased expression of the vascular cell adhesion molecule 1 (VCAM-1), and higher number of CD8^+^ T cells recruited to the lungs. To understand whether such protective effects seen in mice treated with DABK were due to the activation of the B1 receptor in the tumor cells or in the host, wild-type (WT) and B1^−/−^ mice were treated with DABK. The presence of lung metastatic colonies was not significantly different in WT and B1^−/−^ mice both treated with DABK; however, higher VCAM-1 expression and lower CD8^+^ T cells were observed in lungs of B1^−/−^ animals. These results suggest that activation of kinin B1 receptor in the host has an important role in the immune response against the tumor, whereas activation of the B1 receptor exclusively in the tumor cells might be responsible for the reduced metastatic colony establishment.

## Materials and Methods

### Animal Care and Ethics Statement

All methods were performed in accordance with the guidelines of the Brazilian College of Animal Experimentation (COBEA), and all experimental protocols were approved by the Commission of Ethics in Animal Research (CETEA) from the Ribeirao Preto Medical School, University of São Paulo (CETEA, protocol 003/2011).

B1^−/−^ mice and control littermates in a C57BL/6 genetic background were obtained from the Department of Biophysics, Federal University of Sao Paulo, Brazil. The mice were bred and housed in a specific pathogen-free facility with the room temperature controlled at 25°C in a 12-h light/dark cycle; mice received food and water *ad libitum*. Euthanasia was conducted by cervical dislocation.

### Cell Culture

C57BL/6 mice-derived B16F10 cells were cultured in Ham’s F10 media at pH 6.9 supplemented with 10% fetal bovine serum (FBS) and 10 μg/ml of gentamicin. All the experiments were performed when cells were 80–90% confluent.

### Gene Expression Analyses—Semiquantitative and Quantitative PCR

Total RNA was extracted with the TRIzol reagent from 1 × 10^6^ B16F10 cells or 100 mg of tissue sample following manufacturer instructions (Invitrogen). One microgram of total RNA was used for DNase treatment and subsequent reverse transcription using the ImProm-II protocol (Promega). For semiquantitative PCR, target genes were amplified using 100 ng of DNA and Taq platinum DNA polymerase (Invitrogen) following the manufacturer protocol. Samples were loaded in a 2.0% agarose gel stained with SYBR Safe (Invitrogen). For quantitative real-time PCR, we used 10–50 ng of complementary DNA (cDNA) and platinum SYBR Green qPCR SuperMix UDG with ROX reference dye (Invitrogen); results were analyzed using the ABI Prism 7000 sequence detection system. Transcripts were quantified relative to the housekeeping gene beta-actin using the Ct method (Livak and Schmittgen, 2001). The oligonucleotide primers used in the PCR analyses are listed in **Tables S1** and **S2**.

### ERK1/2 Activation

In a six-well plate, 3 × 10^5^ B16F10 cells/well were seeded and incubated for 24 h. Cell culture medium was replaced by fresh medium without FBS, and cells were incubated for 16 h. Cells were stimulated with vehicle or DABK 1 μM for 10, 30, 60, 120, and 180 min. Subsequently, cells were placed in ice, and proteins were extracted to perform western blotting using phospho and total ERK1/2 antibodies (Santa Cruz Biotechnology, sc-7383, and Cell Signaling, 4695, respectively). Densitometry of the bands was quantified using Image J software.

### Confocal Microscopy

Inside 24-well plates, 1 × 10^5^ B16F10 cells were seeded in coverslips and incubated for 24 h. Cell culture medium was replaced by fresh medium without FBS, and cells were incubated for 16 h. Cells were stimulated with vehicle or DABK 1 μM for 24 h. For cells treated with the B1 receptor antagonist, des-Arg^9^-[Leu^8^]-bradykinin (DALBK), treatment with 10 µM of DALBK was performed 30 min prior to adding DABK 1 μM. To access the cytoskeleton morphology, cells were stained with β-tubulin (Sigma, T4026) and phalloidin (Abcam, ab176759). Coverslips were removed from the wells and mounted in slides using *Prolong Antifade kit* (Molecular Probes). Cells were observed and documented in a confocal microscope, Leica TCS SP5.

### Metastasis Induction

Pulmonary melanoma metastasis was induced by injecting 2.5 × 10^5^ B16F10 melanoma cells in 100 µl of phosphate-buffered saline (PBS) in the mice tail vein. Five days after B16F10 cells injection, an osmotic pump containing vehicle or DABK was implanted. Animal weight was monitored daily for 15 days. At the study endpoint, animals were euthanized, and lungs were collected. Samples were either fixed in a 10% formaldehyde solution for histological analysis or immediately frozen at −80°C for molecular analysis.

### Implantation of Mini Pump for DABK Administration

Mice were anesthetized with isoflurane (5% for induction and 2–2.5% for maintenance) diluted in 100% oxygen. The anesthetic mixture was provided *via* an isoflurane vaporizer (Calibrated Vaporizer Takaoka, model ISOVAPOR 1224, K. Takaoka, Brazil), which delivered the anesthetic mixture into a mask. Prior to implantation, mini pumps were prefilled with DABK (Tocris) or vehicle (sterile NaCl 0.9%) and primed by incubation under isotonic saline solution at 37°C for 48 h, according to manufacturer instructions. Under anesthesia, an osmotic mini pump model 1002 (ALZET osmotic pumps) was implanted subcutaneously below the scapular region. DABK was subcutaneously supplied at 1 mg/kg/day for 15 days. We chose a dose of 1 mg/kg/day of DABK and 2 weeks of treatment based on previous publications using the B2 receptor agonist, bradykinin, and the B2 receptor antagonist, SSR240612 (Mori et al., 2008; Qu et al., 2015). Local experts were also consulted to ensure adequate stimulation and to avoid mortality due to drug toxicity.

After the implantation, animals were released from the anesthesia and placed in an individual cage for recovery.

### Histopathological Analyses

Formalin-fixed paraffin-embedded samples were sectioned into 4-μm slices and stained with hematoxylin and eosin. Slides were analyzed using a Leitz Aristoplan microscope coupled to a Leica model DFC280 color camera (Heerbrugg, Switzerland). Melanoma colonies in the lungs were visualized at magnifications of 100× and 200× to observe tissue structure organization. Vessels were visualized at magnification of 200× and counted across 10 random, non-coincident microscopy fields.

### Immunohistochemistry

Formalin-fixed paraffin-embedded samples were sectioned into 4-μm slices. Deparaffinized sections were subjected to antigen retrieval with citrate solution, pH 6.0, and treated with 3% hydrogen peroxide to inhibit endogenous peroxidase activity. After blocking slides with FBS, sections were incubated overnight at 4°C with a 1:200 dilution of anti-vascular endothelial growth factor (VEGF) (Abcam, ab1316), 1:100 dilution of anti-VCAM-1 (Santa Cruz Biotechnology, sc-1504), anti-CD4 (Abcam, ab51312), and anti-CD8 (Santa Cruz Biotechnology, sc-7970) monoclonal antibody. Slides were treated with biotin-conjugated secondary antibody (Dako) and with horseradish peroxidase-conjugated avidin (Dako). Peroxidase activity was localized for all samples with 3,3′-diaminobenzidine, counterstained with hematoxylin, dehydrated, cleared, and mounted in mounting medium Entellan (Merck).

### Image Quantification

Evaluation of VEGF, VCAM-1, and the presence of CD4^+^ and CD8^+^ T cells was performed by optical density in the image analysis using Leica QWin software (Leica Microsystems Image Solutions, UK) in conjunction with a Leica DMR microscope (Leica, Microsystems, Germany), video camera (Leica Microsystems, Switzerland), and an online computer. The thresholds for positive staining were established for each slide after enhancing the contrast to a point at which the cells were easily identified as a brown staining. Ten randomly chosen, non-coincident fields were measured for each group at 200× across a total of 2.3-mm^2^ area.

### Statistical Analysis

Statistical analysis was performed using Student’s *t*-tests. For body weight comparison, we performed a two-way ANOVA test with Geisser–Greenhouse correction. Data are expressed as mean ± SEM. Differences between mean values were considered significant when *p* < 0.05.

## Results

### Kinin B1 Receptor Is Constitutively Expressed in B16F10 Melanoma Cells and Functionally Activated by Its Agonist DABK

**Figure 1:**
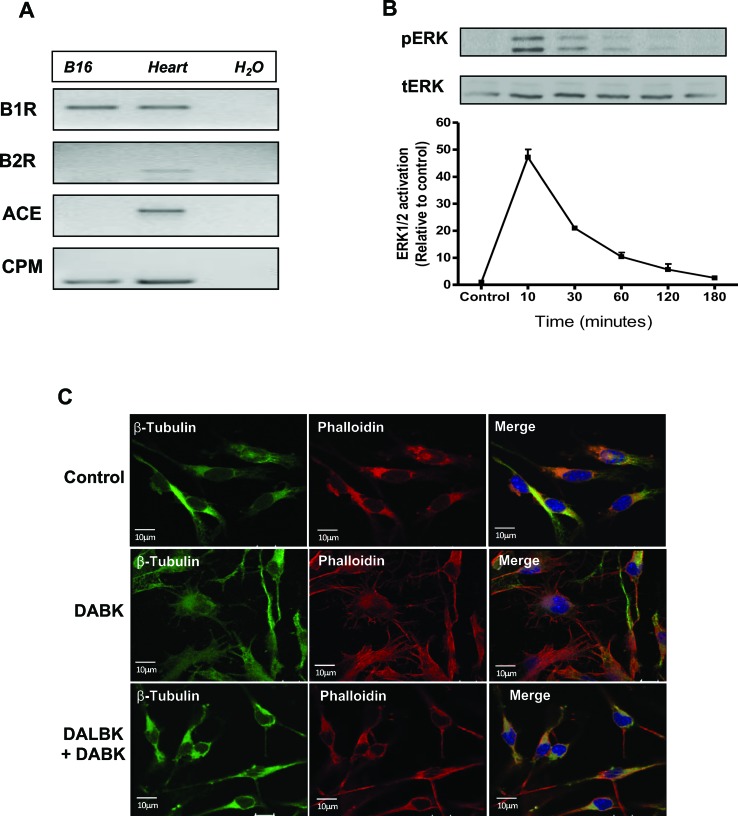
Kinin B1 receptor is expressed in B16F10 cells and responds to DABK stimulus. **(A)** mRNA expression of kinin B1 and B2 receptors, angiotensin-converting enzyme (ACE), and carboxypeptidase M (CPM) in B16F10 cells assessed by semiquantitative PCR. Positive control: mRNA from mice heart. Negative control: H_2_O. **(B)** Kinetics of ERK1/2 phosphorylation after B16F10 cells stimulation with DABK 1 μM. Upper panel: representative western blotting of two independent experiments. **(C)** Microtubule (β-tubulin) and actin filament (phalloidin) staining in B16F10 cells treated with vehicle (control), DABK 1 μM, and DALBK 10 μM. Nuclei were stained with DAPI (blue). DABK, des-Arg^9^-bradykinin; DALBK, des-Arg^9^[Leu^8^]-bradykinin; DAPI, 4′,6-diamidino-2-phenylindole; mRNA, messenger RNA.

Endogenous expression of B1 receptor and other key components of the kallikrein–kinin system, such as angiotensin-converting enzyme (ACE) and carboxypeptidase M (CPM), was evaluated. As shown in our previous work (Dillenburg-Pilla et al., 2013), B1 receptor is constitutively expressed in B16F10 cells while messenger RNA (mRNA) expression of B2 receptor is not detected, providing a good model to study the effects of the B1 receptor activation. Here, we also show that CPM is constitutively expressed, while ACE is not detected (**Figure 1A**). ERK1/2 phosphorylation kinetics in B16F10 cells after DABK stimulation show a typical GPCR activation profile (Kumari et al., 2019) (**Figure 1B**). In addition, after stimulation with DABK, B16F10 cells presented changes in morphology as seen by the modification in the actin and tubulin expression pattern. This effect was impaired when B16F10 cells were pre-incubated with B1 receptor antagonist DALBK, followed by DABK stimulation (**Figure 1C**).

### Systemic Treatment With Kinin B1 Receptor Agonist Reduces Incidence of B16F10 Metastatic Colonies in Lungs of Mice

**Figure 2:**
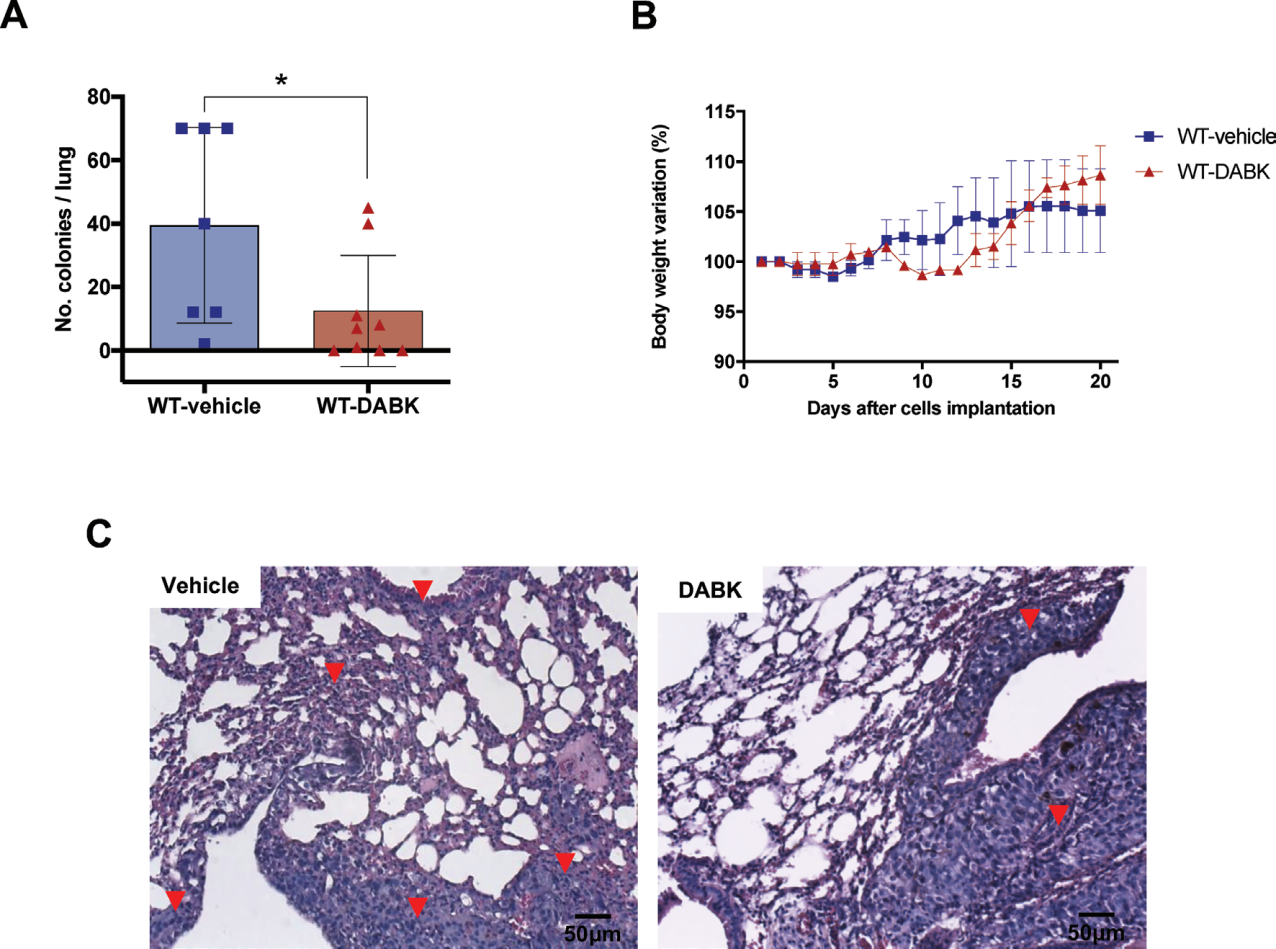
Des-Arg^9^-bradykinin (DABK) treatment decreases metastatic melanoma colony establishment in lungs of mice. Lung metastasis was induced by the injection of B16F10 cells in the tail vein of mice, and after 5 days of cells injection, wild-type (WT) mice received vehicle or DABK (1 mg/kg/day) for 15 days. **(A)** Number of detectable melanoma colonies established in the lungs of animals treated with vehicle (control) or DABK (control: *n* = 7, DABK: *n* = 9, *p = 0.04). **(B)** Body weight variation of mice after receiving B16F10 cells in the tail vein. **(C)** Histological images showing the organization of melanoma colonies established in mice lungs. ▼ indicates the presence of small metastatic melanoma colonies established in the lungs of mice.

We investigated whether activation of kinin B1 receptor could modulate metastatic melanoma growth in the lungs. Melanoma metastasis was induced by delivering B16F10 cells directly into the blood flow, and 5 days after cells injection, animals were treated with vehicle or DABK for 2 weeks. At the end of the protocol, lung metastases were screened. Animals treated with DABK presented a lower number of metastatic colonies as compared with animals treated with vehicle (**Figure 2A**), corroborating the previous findings that kinin B1 receptor has a positive role protecting the host from metastatic melanoma colony establishment (Dillenburg-Pilla et al., 2013; Maria et al., 2016). Differences in gain or loss of weight observed during the period studied were not statistically significant (**Figure 2B**). Given that colony morphology and characteristics are very important features for metastasis aggressiveness (Chillà et al., 2018), we histologically assessed the lungs and found the prevalence of a higher number of small metastatic colonies established in mice that did not receive DABK treatment as compared to colonies developed in animals that received DABK treatment (**Figure 2C**).

### DABK Treatment Decreases VCAM-1 Expression in Mice Bearing Melanoma Metastasis in Lungs

**Figure 3:**
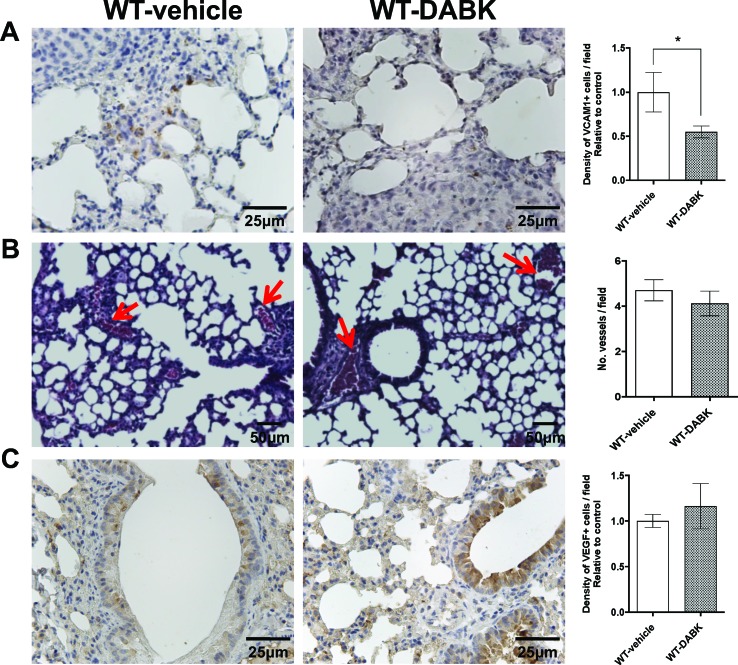
Effects of des-Arg^9^-bradykinin (DABK) treatment in vascular cell adhesion molecule 1 (VCAM-1) expression and vascularization in lungs of mice with metastatic melanoma. Lung metastasis was induced by the injection of B16F10 cells in the tail vein of mice, and after 5 days of cells injection, wild-type (WT) mice received vehicle or DABK (1 mg/kg/day) for 15 days. **(A)** VCAM-1-positive cells in metastatic lungs from mice that received DABK or vehicle (*p = 0.03, *n* = 6). Left panel: representative images, right panel: quantification of positive expression ± SEM, relative to WT-vehicle in 10 different high magnification fields. **(B)** Left: histological images showing vessels (red arrows) in lungs after 20 days of B16F10 cells injection. Right: quantification of number of vessels per field. Data are expressed as number of vessels ± SEM (control: *n* = 7, DABK: *n* = 9, *p* = 0.43). **(C)** Vascular endothelial growth factor (VEGF)-positive cells in metastatic lungs from mice that received DABK or vehicle (*p* = 0.51, *n* = 6). Left panel: representative images, right panel: quantification of positive expression ± SEM, relative to WT-vehicle in 10 different high magnification fields.

Melanomas have a high potential to metastasize to distant sites (Lau et al., 2015), and its spreading is associated with the efficient intravasation in the circulation to eventually colonize a distant site (Scott and Harpole, 2016). We compared expression of VCAM-1 in lungs of animals treated with vehicle or DABK, as VCAM-1 plays an important role in the metastatic process mediating tumor cell adhesion to the endothelium (Tichet et al., 2015). Animals treated with DABK presented lower VCAM-1 expression in the lungs (**Figure 3A**). VEGF is considered one of the most important pro-angiogenic factors, and it has been extensively studied as a prognostic marker for angiogenesis (Kim, 2013; Ponert et al., 2018). No significant differences in the number of vessels were found in the lungs of mice treated with vehicle or DABK (**Figure 3B**), and corroborating these data, we also did not see differences in VEGF expression in the lungs of both groups (**Figure 3C**).

### DABK Recruits CD8+ T Cells to Lungs With Metastatic Melanoma and Decreases Pro- and Anti-Inflammatory Cytokines

**Figure 4:**
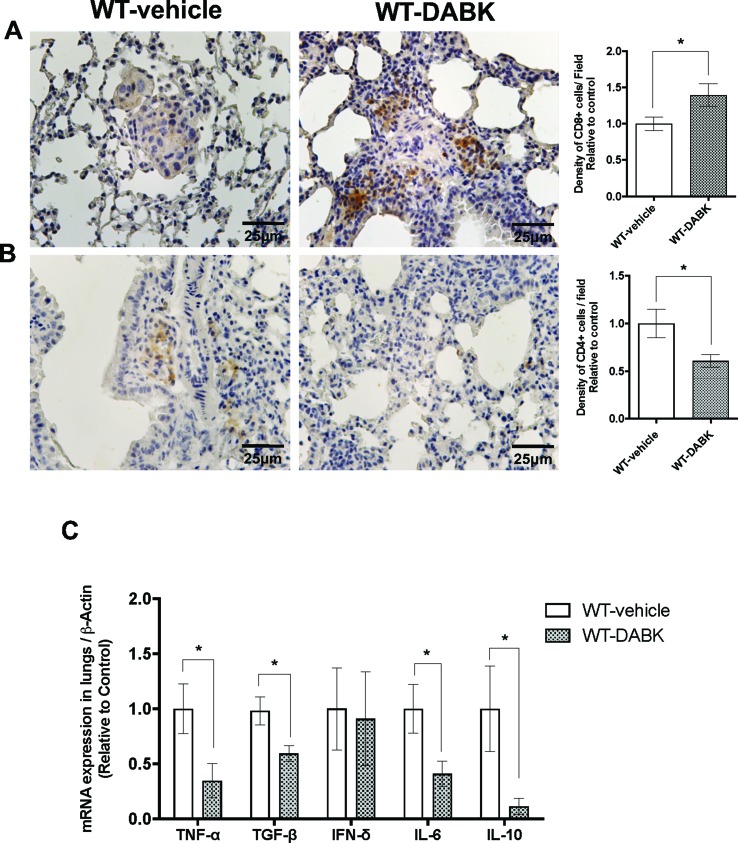
Effects of des-Arg^9^-bradykinin (DABK) treatment in the recruitment of CD4^+^ and CD8^+^ T cells to lungs of mice with metastatic melanoma. Lung metastasis was induced by the injection of B16F10 cells in the tail vein of mice, and after 5 days of injection, wild-type (WT) mice received vehicle or DABK (1 mg/kg/day) for 15 days. **(A)** CD8^+^ T cells in metastatic lungs from mice that received DABK or vehicle (*p = 0.021, *n* = 6). Left panel: representative images, right panel: quantification of optical density of positive cells ± SEM, relative to WT-vehicle in 10 different high magnification fields. **(B)** CD4^+^ T cells in metastatic lungs from mice that received DABK or vehicle (*p = 0.026, *n* = 6). **(C)** Cytokine messenger RNA (mRNA) profile in melanoma metastatic lungs. Experiments were performed by quantitative real-time PCR. Data are displayed as fold of change from relative expression of target gene/β-actin ± SEM using the delta-delta Ct method (*n* = 7, *p < 0.05).

CD4^+^ and CD8^+^ T cells are key players of the immune process protecting the host against tumors. As melanoma is very immunogenic (Haanen, 2013), we evaluated the presence of CD4^+^ and CD8^+^ T cells in metastatic lungs of animals treated with vehicle or DABK. We found decreased expression of CD4^+^ T cells and increase of CD8^+^ T cells in the lungs of DABK-treated animals (**Figures 4A, B**). To further investigate whether the activation of B1 receptor would also modulate inflammatory responses in metastatic lungs, we evaluated mRNA expression of pro- and anti-inflammatory cytokines in the lungs of animals treated or not with DABK. Our results show that the pro-inflammatory tumor necrosis factor (TNF)-α and interleukin (IL)-6 and anti-inflammatory cytokines transforming growth factor (TGF)-β and IL-10 were downregulated upon DABK treatment (**Figure 4C**), suggesting that activation of the B1 receptor by DABK might be playing a dual role, anti- and pro-inflammatory, under the progress of metastasis in both groups. Although the mechanisms are yet not well understood, we could observe that activation of B1 receptor influences the decrease of CD4^+^ cells and alters the T-helper cytokine profile, supporting the key role of the B1 receptor in inflammation.

### Knockout of B1 Receptor in Mice Abolishes the DABK Effect on Colony Establishment

**Figure 5:**
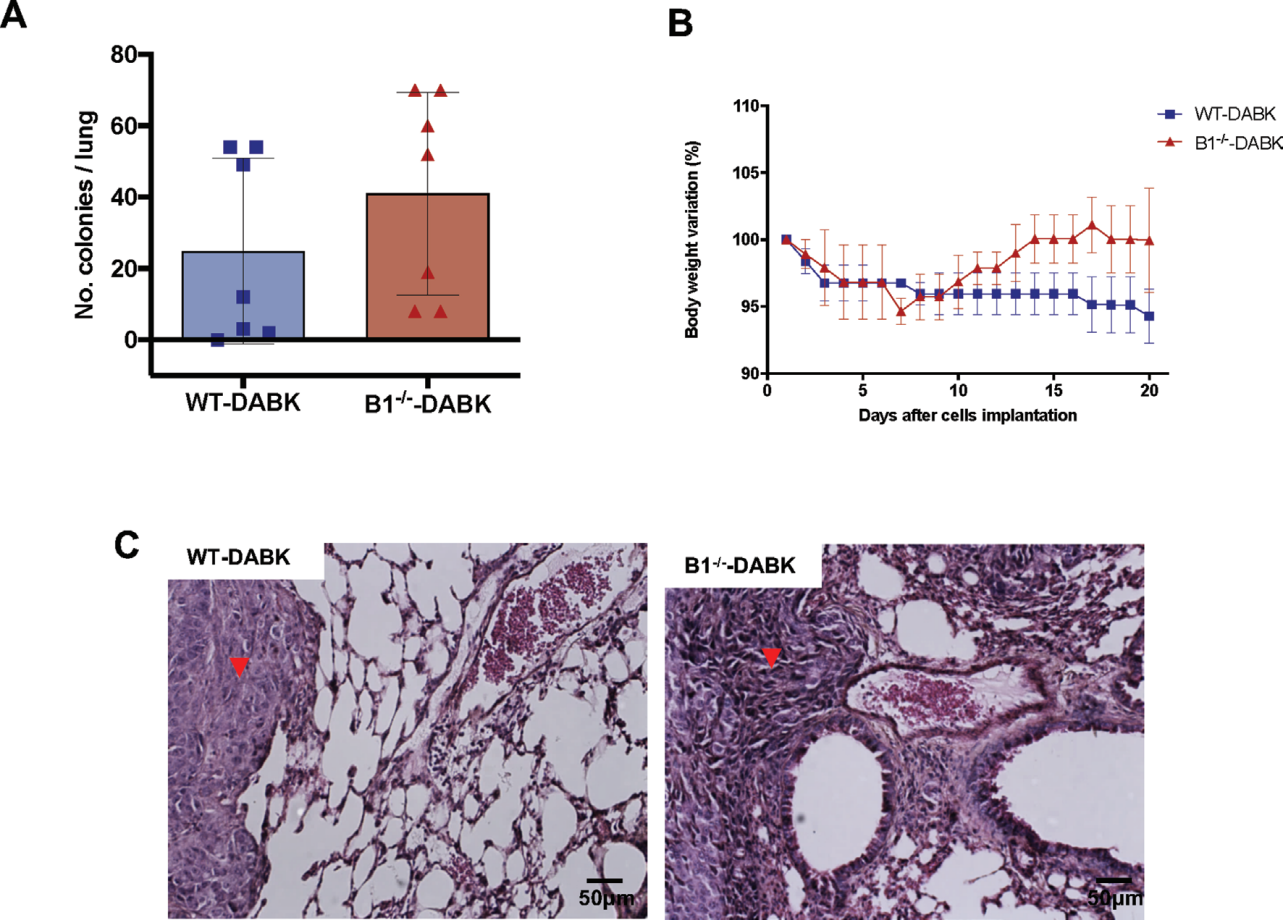
Activation of the B1 receptor in the microenvironment does not alter the number of colonies established. Lung metastasis was induced by the injection of B16F10 cells in the tail vein of mice, and after 5 days of cells injection, mice received des-Arg^9^-bradykinin (DABK) (1 mg/kg/day) for 15 days. **(A)** Number of melanoma colonies established in the lungs of wild-type (WT) and B1 receptor knockout mice (B1^−/−^) treated with DABK (WT: *n* = 7, B1^−/−^: *n* = 7, *p* = 0.28). **(B)** Body weight variation of wild type (WT) and B1 receptor knockout (B1^-/-^) mice after receiving B16F10 cells in the tail vein. **(C)** Histological images showing the organization of melanoma colonies established in mice lungs. ▼ indicates the presence of metastatic melanoma colonies stablished in lungs of mice.

To understand whether the protective effect of DABK treatment is due to activation of the B1 receptor present in the microenvironment or in the tumor cells, we treated WT and B1^−/−^ mice bearing melanoma metastasis with DABK, which allowed us to access the effects B1 receptor activation by DABK, exclusively in the tumor cells. No significant difference was observed in the number of colonies between B1^−/−^ and WT animals treated with DABK (**Figure 5A**). Differences in gain or loss of weight observed in the mice during the period studied were not statistically significant (**Figure 5B**). Histological observation of metastatic lungs showed that melanoma colonies established in DABK-treated B1^−/−^ mice presented less organized structures with infiltrative characteristics as compared to tumor colonies formed in the WT DABK-treated animals (**Figure 5C**).

### Melanoma Metastatic Lungs From Kinin B1^−/−^ Mice Treated With DABK Acquire Increased Number of VCAM-1, Vessels, and VEGF Expression

**Figure 6:**
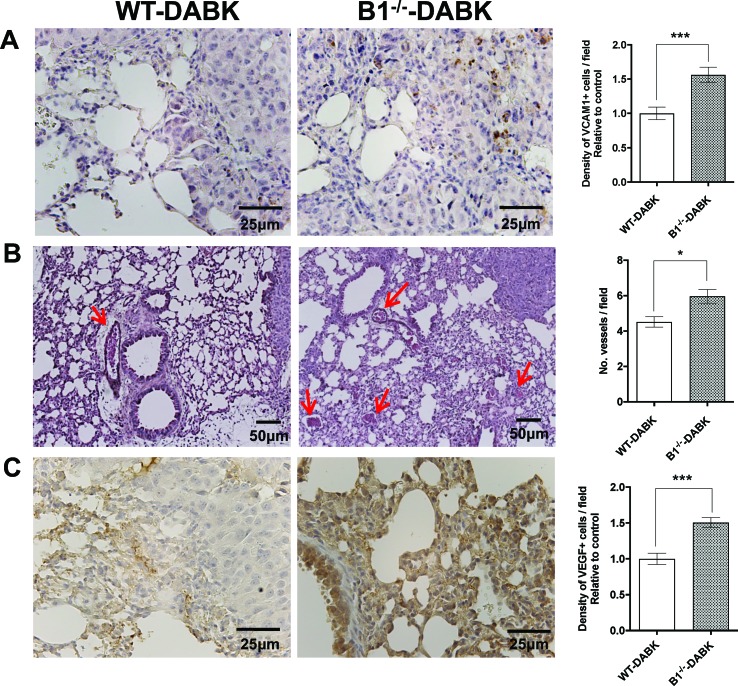
Effects of des-Arg^9^-bradykinin (DABK) treatment in vascularization and vascular cell adhesion molecule 1 (VCAM-1) expression in lungs of wild-type (WT) and B1 receptor knockout mice (B1^−/−^) with metastatic melanoma. Lung metastasis was induced by the injection of B16F10 cells in the tail vein of WT and B1^−/−^ mice, and after 5 days of injection, mice received DABK (1 mg/kg/day) for 15 days. **(A)** VCAM-1-positive cells in metastatic lungs of WT and B1^−/−^ mice that received DABK treatment (***p = 0.0003, *n* = 6). Left panel: representative images, right panel: quantification of positive expression ± SEM, relative to WT-DABK in 10 different high magnification fields. **(B)** Left: histological images showing vessels (red arrows) in lungs after 20 days of B16F10 cells injection. Right: quantification of number of vessels per field. Data are expressed as number of vessels ± SEM (WT: *n* = 7, B1^−/−^: *n* = 7, *p = 0.01). **(C)** Vascular endothelial growth factor (VEGF)-positive cells in metastatic lungs of WT and B1^−/−^ mice that received DABK treatment (***p = 0.0001, *n* = 6).

To investigate if activation of B1 receptor in the microenvironment or in B16F10 cells plays a role in metastatic lung vascularization, lungs of WT and B1^−/−^ mice treated with DABK were compared. We observed increased expression of VCAM-1 (**Figure 6A**), and histological analysis revealed a significant increase in the number of blood vessels in the lungs of B1^−/−^ mice treated with DABK as compared to WT animals also treated with DABK (**Figure 6B**). Corroborating these data, immunohistochemistry analysis showed increased expression of VEGF in lungs of B1^−/−^ DABK-treated mice (**Figure 6C**). Moreover, in our previous study (Maria et al., 2016), we did not observe differences in the number of melanoma colonies established in the lungs of non-treated B1^−/−^ mice compared to non-treated WT animals and found an increased number of vessels in the lungs of B1^−/−^ mice.

### Activation of the Kinin B1 Receptor in the Tumor Microenvironment Contributes to CD8+ T Cell Recruitment and Increases IL-6 Expression in Metastatic Lungs

**Figure 7:**
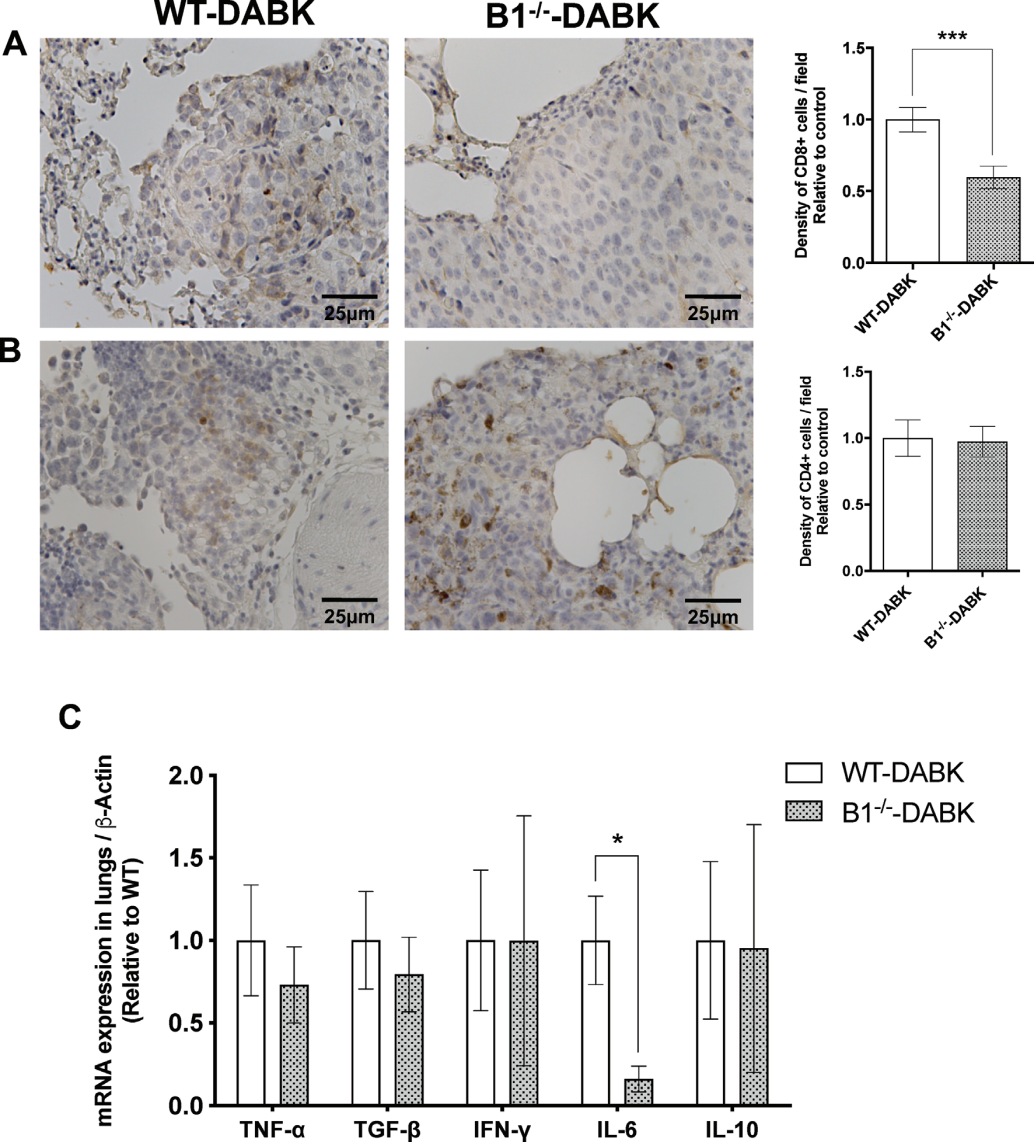
Effects of des-Arg^9^-bradykinin (DABK) treatment in the recruitment of CD4^+^ and CD8^+^ T cells to lungs of wild-type (WT) and B1 receptor knockout mice (B1^−/−^) with metastatic melanoma. Lung metastasis was induced by the injection of B16F10 cells in the tail vein of WT and B1^−/−^ mice, and after 5 days of injection, mice received DABK (1 mg/kg/day) for 15 days. **(A)** CD8^+^ T cells in metastatic lungs from mice that received DABK or vehicle (***p = 0.0008, *n* = 6). Left panel: representative images, right panel: quantification of optical density of positive cells ± SEM, relative to WT-DABK in 10 different high magnification fields. **(B)** CD4^+^ T cells in metastatic lungs from mice that received DABK or vehicle (*p* = 0.88, *n* = 6). **(C)** Cytokine messenger RNA (mRNA) profile in melanoma metastatic lungs. Experiments were performed by quantitative real-time PCR. Data are displayed as fold of change from relative expression of target gene/β-actin ± SEM using the delta-delta Ct method (*n* = 7, *p < 0.05).

To study whether the increased recruitment of CD8^+^ T cells to the tumor area was due to the activation of the kinin B1 receptor in the host or in the tumor cells, we treated WT and B1^−/−^ animals with DABK and observed a significant decrease in the frequency of CD8^+^ T cells in lungs of B1^−/−^ mice (**Figure 7A**). Regarding CD4^+^ T cells, we did not find significant differences between WT and B1^−/−^ animals (**Figure 7B**). We also evaluated cytokine mRNA expression profiles, in which the data show decreased expression of the pro-inflammatory cytokine IL-6 in the lungs of B1^−/−^ mice, suggesting that the activation of the B1 receptor in the host plays a role in mediating pro-inflammatory responses in the tumor microenvironment during melanoma growth (**Figure 7C**).

## Discussion

The KKS is involved in a number of pathophysiological processes such as vasodilation, inflammation, vascular permeability, pain, and diabetes (Kashuba et al., 2013; Liu et al., 2017). Kinins are rapidly generated after tissue injury, playing an important role in the development and control of inflammatory process, and both B1 and B2 kinin receptors are critical in the process of acute and chronic inflammation (Kaplan and Ghebrehiwet, 2010). Recent studies show that inflammation exhibits a paradoxical role in tumor development, either promoting antitumor immune response or supporting tumor growth and metastasis (Jain et al., 2014). Although the B1 receptor is known for its role in the regulation of inflammatory processes (Costa-Neto et al., 2008), the precise function of kinin B1 receptor in tumor development and metastasis is still controversial. Previous work from our group showed that either the presence of the B1 receptor in the host microenvironment or pretreatment of melanoma cells with the B1 receptor agonist can protect against tumor progression (Dillenburg-Pilla et al., 2013; Maria et al., 2016).

In the current study, we treated C57BL/6 mice bearing melanoma metastasis in the lungs with a B1 receptor agonist, DABK, to investigate whether systemic activation of the B1 receptor would be efficient to improve antitumor response. Our results showed that mice treated during 2 weeks with DABK had a reduced number of detectable melanoma colonies and increased recruitment of CD8^+^ T cells in the lungs when compared to non-treated animals. Moreover, CD4^+^ T cell infiltration was decreased, resulting in a lower CD4^+^/CD8^+^ ratio in the lungs of animals treated with DABK. It is known that the presence of T cells in tumor tissue may impact the prognosis of the disease (Kluger et al., 2015; Falkenius et al., 2017) and that a lower CD4^+^/CD8^+^ ratio is correlated with less aggressive tumors (Fortis et al., 2017). In fact, CD8^+^ T cells are recruited to the tumor area acting as tumor killers (Hadrup et al., 2013) and are also key components in the maintenance of tumor latency. As treatment with DABK was shown to increase the number of CD8^+^ T cells in the metastatic lungs, we hypothesize that activation of B1 receptor by DABK could be playing a role in promoting an increase of the immune response against the tumor. VCAM-1 has been shown to be involved in immune evasion (Lin et al., 2007; Wu, 2007) and allows a strong interaction of its ligand VLA4 on monocytes and tumor-associated macrophages, recruiting them to metastatic lungs (Schlesinger and Bendas, 2015). We observed that animals treated with DABK presented a significant decrease of VCAM-1 expression in metastatic lungs when compared with non-treated animals. These data reinforce our hypothesis that B1 receptor might be an important mediator of immune response against metastatic melanoma establishment. The role of B1 receptor in immune cell migration has been previously described (Pesquero et al., 2000;Estrela et al., 2014).

Further, we sought to understand whether such beneficial effects of DABK treatment observed were due to B1 receptor activation in the host or in the tumor cells. To answer this question, WT and B1^−/−^ mice bearing melanoma metastasis were treated with DABK. We found reduced CD8^+^ T cell recruitment in the lungs of B1^−/−^ mice treated with DABK compared to treated WT mice. In a previous study, we also observed a decreased number of CD8^+^ T cells in the metastatic lungs of B1^−/−^ mice compared to WT when none of the groups were treated with DABK, reinforcing the hypothesis that activation of the B1 receptor in the microenvironment plays a role in the immune response against melanoma by recruiting CD8^+^ T cells to the metastatic area. In this same study, the number of colonies detected in non-treated WT or B1^−/−^ mice was not statistically significant; however, the diameter of colonies developed in B1^−/−^ was shown to be significantly larger (Maria et al., 2016). In accordance with previous data, in the current study, when both WT and B1^−/−^ mice were treated with DABK, differences in the number of colonies established in the lungs were not statistically significant, but increased number of vessels and VEGF expression in B1^−/−^ mice were observed. These data suggest that the absence of the B1 receptor in the microenvironment favors the onset of neovascularization in metastatic lungs. Nevertheless, it is important to take into consideration that the B1 receptor is described to promote angiogenesis when activated by its agonists (Emanueli et al., 2002;Colman, 2006). However, the effect of the B1 receptor is not as well described as that of the B2 receptor and its pro-angiogenic role (Sanchez de Miguel et al., 2008;Terzuoli et al., 2018). As it is already known that deletion of the B1 receptor in mice upregulates B2 receptor expression (Marcon et al., 2013; Rodrigues et al., 2013), the increased vascularization and VEGF expression in lungs of B1^−/−^ mice treated with DABK could also be due to an indirect effect of B2 receptor in order to overcome the absence of B1 receptor. Concerning the effects of B2 receptor activation by DABK in the tumor cells, the hypothesis of an off target can be ruled out as the expression of the B2 receptor is not detected in B16F10 cells. However, further studies are necessary to clarify whether the B2 receptor is involved in the mechanisms of metastasis establishment in B1^−/−^ animals.

With the development of immunotherapy approaches, overall survival of patients with melanoma has increased in the last few years. However, mechanisms of resistance to different types of therapies are still one of the major challenges for treatment. Thus, development of effective tools to act alone or in combination with adjuvant therapies is important to overcome melanoma resistance. The B1 receptor belongs to the family of GPCRs. GPCRs are known to be the largest family of cell surface receptors that regulate different biological responses and, therefore, are frequently used as drug targets for several diseases, including hypertension and cancer therapy (Brinks and Eckhart, 2010; Liu et al., 2016). The protective effect of the B1 receptor against melanoma has been already described (Dillenburg-Pilla et al., 2013; Maria et al., 2016). However, the effect of the B1 receptor agonist treatment as a therapy and whether the protective effects are due to B1 receptor activation in tumor cells or in the host were not clear. Here, we show that systemic activation of B1 receptor by its agonist, DABK, decreased metastatic colony establishment in lungs compared to non-treated animals. However, this effect is not detectable when both WT and B1^−/−^ mice are treated with DABK, suggesting that the main effect of metastatic colony establishment and growth is driven by the B1 receptor activation in tumor cells and not by the receptor in the host. Furthermore, we believe that the immune response against the tumor may occur by a dual effect of B1 in tumor cells and host immune response, since we observed alterations in the recruitment of CD8^+^ T cells and VCAM-1 expression in the lungs of mice that had the B1 receptor activated either in the microenvironment or in the tumor cells. Taken together, more studies are necessary to elucidate the mechanisms in which the activation of the B1 receptor *via* its agonist may act to contribute to the impairment of metastasis advance. However, the present study brings important insights to explore novel approaches for drug resistance and metastatic melanoma treatment.

## Data Availability

All datasets generated for this study are included in the manuscript/**Supplementary Files**.

## Ethics Statement

The animal study was reviewed and approved by Commission of Ethics in Animal Research (CETEA) from the Ribeirao Preto Medical School, University of São Paulo (CETEA, protocol 003/2011).

## Author Contributions

Conception and design of the study: AGM, CN, and CC-N. *In vitro* assays: AGM and AOM. *In vivo* assays: AGM and MTD. Molecular and histopathological analysis: AGM, PD-P, EF, and SGR. B1 receptor knockout mice generation: JBP. Study supervision: CC-N. Manuscript drafting: AGM, CN, and CC-N.

## Funding

This work was supported by the Sao Paulo State Research Foundation (FAPESP grants 2010/13346-4 and 2012/20148-0); AGM received a PhD scholarship from FAPESP (2011/02144-4).

## Conflict of Interest Statement

The authors declare that the research was conducted in the absence of any commercial or financial relationships that could be construed as a potential conflict of interest.
