# Supplementary material for: Activation of the Kinin B1 Receptor by Its Agonist Reduces Melanoma Metastasis by Playing a Dual Effect on Tumor Cells and Host Immune Response
Source: Front Pharmacol. 2019 Sep 25;10:1106. doi: 10.3389/fphar.2019.01106 (PMC6774293; doi:10.3389/fphar.2019.01106)
Supplement: Supplementary file 1 [file Table_1.docx]

**Supplementary Table S1:** Sequence of primers and conditions used in semi-quantitative PCR

| **Gene** | **pb** | **Tm**  **(°C)** | **Forward (5’ - 3’)** | **Reverse (5’ - 3’)** |
| --- | --- | --- | --- | --- |
| **B1 Receptor** | 291 | 55 | 5’ CACGAAGCTTGGCACTTTGT 3’ | 5’ GTCTGTGAGCTCCTTCCAGAA 3’ |
| **B2 Receptor** | 341 | 56 | 5’ GCACTGTGGCCGAGATCTA 3’ | 5’ GCTGTATTCCCTCATGGTCCT 3’ |
| **ACE** | 500 | 55 | 5’ ACTGAAGACCCCCCA ACG 3’ | 5’ GGAACGCCACACACATGT T 3’ |
| **CPM** | 141 | 55 | 5’ AAACATTTGTCCTCTCTG CG 3’ | 5’ TGTAGGCCAGGTGTTGGAAA 3’ |

pb: base pairs; Tm: melting temperature; ACE: angiotensin converting enzyme; CPM: Carboxypeptidase M.

**Supplementary Table S2:** Sequence of primers and conditions used in Real-Time quantitative PCR

| **Gene** | **pb** | **Tm**  **(°C)** | **Forward (5’ - 3’)** | **Reverse (5’ - 3’)** |
| --- | --- | --- | --- | --- |
| **IL-6** | 280 | 55 | 5’ CATCCAGTTGCCTTCTTGGG 3’ | 5’ CCAGTTTGGTAGCATCCATC 3’ |
| **IL-10** | 193 | 55 | 5’GGTTGCCAAGCCTTATCGGAAATGA 3’ | 5’ TTCACCTGCTCCACTGCCTTGCT 3’ |
| **TNF-α** | 140 | 55 | 5’ AAGCCTGTAGCCCACGTCGTA 3’ | 5’ AGGTACAACCCATCGGCTGG 3’ |
| **TGF-β** | 94 | 55 | 5’ GCAACATGTGGAACTCTACCA G 3’ | 5’ CAGCCACTCAGGCGTATCA 3’ |
| **IFN-γ** | 179 | 55 | 5’ CAGCAACAGCAAGGCGAAAAAGG 3’ | 5’ AATCTCTTCCCCACCCCGAATCA 3’ |
| **β-Actin** | 125 | 55 | 5’ ATCATGAAGTGTGACGTTGACA 3’ | 5’CACTGTGTTGGCATAGAGGTCT 3’ |

pb: base pairs; Tm: melting temperature.
